# Functionalized Calcium Carbonate-Based Microparticles as a Versatile Tool for Targeted Drug Delivery and Cancer Treatment

**DOI:** 10.3390/pharmaceutics16050653

**Published:** 2024-05-13

**Authors:** Lara Biny, Evgeniia Gerasimovich, Alexander Karaulov, Alyona Sukhanova, Igor Nabiev

**Affiliations:** 1 Université de Reims Champagne-Ardenne, BIOSPECT, 51100 Reims, France; lara.biny@univ-reims.fr; 2 Life Improvement by Future Technologies (LIFT) Center, Laboratory of Optical Quantum Sensors, Skolkovo, 143025 Moscow, Russia; ewgenia-gerasimowitch@yandex.ru; 3 Laboratory of Nano-Bioengineering, National Research Nuclear University MEPhI (Moscow Engineering Physics Institute), 115409 Moscow, Russia; 4 Department of Clinical Immunology and Allergology, Institute of Molecular Medicine, Sechenov First Moscow State Medical University (Sechenov University), 119146 Moscow, Russia; drkaraulov@mail.ru

**Keywords:** calcium carbonate, microparticles, microcapsules, core/shell structures, targeted delivery, anticancer treatment

## Abstract

Nano- and microparticles are increasingly widely used in biomedical research and applications, particularly as specific labels and targeted delivery vehicles. Silica has long been considered the best material for such vehicles, but it has some disadvantages limiting its potential, such as the proneness of silica-based carriers to spontaneous drug release. Calcium carbonate (CaCO_3_) is an emerging alternative, being an easily available, cost-effective, and biocompatible material with high porosity and surface reactivity, which makes it an attractive choice for targeted drug delivery. CaCO_3_ particles are used in this field in the form of either bare CaCO_3_ microbeads or core/shell microparticles representing polymer-coated CaCO_3_ cores. In addition, they serve as removable templates for obtaining hollow polymer microcapsules. Each of these types of particles has its specific advantages in terms of biomedical applications. CaCO_3_ microbeads are primarily used due to their capacity for carrying pharmaceutics, whereas core/shell systems ensure better protection of the drug-loaded core from the environment. Hollow polymer capsules are particularly attractive because they can encapsulate large amounts of pharmaceutical agents and can be so designed as to release their contents in the target site in response to specific stimuli. This review focuses first on the chemistry of the CaCO_3_ cores, core/shell microbeads, and polymer microcapsules. Then, systems using these structures for the delivery of therapeutic agents, including drugs, proteins, and DNA, are outlined. The results of the systematic analysis of available data are presented. They show that the encapsulation of various therapeutic agents in CaCO_3_-based microbeads or polymer microcapsules is a promising technique of drug delivery, especially in cancer therapy, enhancing drug bioavailability and specific targeting of cancer cells while reducing side effects. To date, research in CaCO_3_-based microparticles and polymer microcapsules assembled on CaCO_3_ templates has mainly dealt with their properties in vitro, whereas their in vivo behavior still remains poorly studied. However, the enormous potential of these highly biocompatible carriers for in vivo applications is undoubted. This last issue is addressed in depth in the Conclusions and Outlook sections of the review.

## 1. Introduction

Microparticles are widely used in various fields of research and drug delivery applications [1,2]. Among the various materials used for microparticle fabrication, silica has long been considered the best candidate, but it has several disadvantages that limit its clinical potential, especially in preventing spontaneous drug release [3]. Calcium carbonate (CaCO_3_) is an abundant, inexpensive, biocompatible material with suitable chemical and physical properties, such as a small size of particles with a large surface area [4]. These properties make it an attractive material for numerous biomedical applications and an ideal choice for targeted cancer immunotherapy [5]. There are three polymorphs of CaCO_3_ particles: calcite, aragonite, and vaterite crystals. Though less thermodynamically stable than the others, vaterite crystals are spherical, composed of nanodomains, and highly porous, which makes them a good candidate for use in drug delivery systems [6,7].

The most common methods of synthesis of CaCO_3_ microparticles are solid–liquid–gas carbonation [8] and chemical precipitation through the reaction of CaCl_2_ with Na_2_CO_3_ in an aqueous medium [9]. There are also other methods of synthesis of CaCO_3_ microparticles [10], such as supercritical fluid technology [11] and emulsion techniques [12,13]. In the course of synthesis, the temperature, pH, reagent concentrations, and other parameters can be controlled to optimize the size, morphology, and composition of the microparticles. It has been shown that the gradual addition of a calcium nitrate solution to the sodium carbonate solution allows controlling the saturation of the reaction medium and obtaining smaller CaCO_3_ particles after prolonged agitation. Overall, temperature influences particle morphology and polymorphism, whereas the calcium and carbonate ion concentrations determine their size [14]. These different techniques of synthesis offer flexible approaches for obtaining CaCO_3_ particles suitable for various therapeutic applications. CaCO_3_-based microparticles have a wide range of potential applications, particularly in targeted drug delivery. Their use can offer significant advantages in terms of efficiency, cost-effectiveness, and sustainability compared to existing materials.

**Figure 1:**
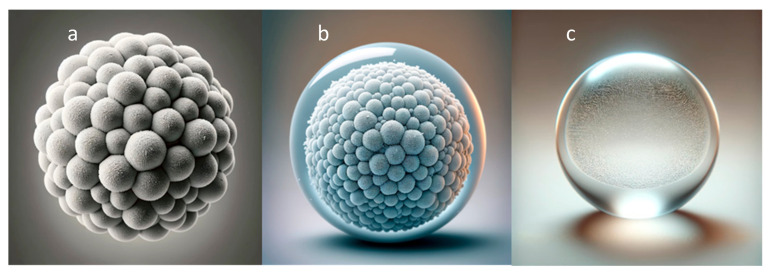
Spherical CaCO_3_-based microparticles for targeted cancer therapy: (**a**) a CaCO_3_ core-only microparticle; (**b**) a CaCO_3_ core/shell microparticle; (**c**) a polyelectrolyte shell-only microcapsule without a core.

Three main types of CaCO_3_-based microparticles with sizes ranging from about 0.2 to 6 µm have been extensively studied: core-only microparticles, polymer-coated cores (or core/shell microparticles), and hollow (shell) polymer capsules, for which CaCO_3_ particles are used as sacrificial templates [2,15,16] (Figure 1). Each of these types possesses unique characteristics suitable for specific applications in cancer treatment.

Core-only microparticles are primarily used due to their capacity for absorbing and carrying therapeutic agents. Their simple, porous structure also ensures drug release. However, their use is limited by their lack of targeting specificity and insufficient resistance to the potentially aggressive factors of the biological microenvironment. Additional strategies may be necessary to prevent their degradation or aggregation during the delivery [17].

Core/shell structures are considerably more advantageous because their polyelectrolyte shell provides enhanced protection of the encapsulated compound compared to core-only systems and can be functionalized to ensure specific targeting. Current research focuses on developing new strategies to enhance stability, targeting, and release control by coating microparticles with polymers [18] or lipids [19]. These microparticles can be designed to respond to specific stimuli, such as changes in pH [20,21] or temperature [22], by releasing their contents. They are commonly fabricated by means of layer-by-layer (LbL) deposition of alternating anionic and cationic polyelectrolytes, depending on the charge of the template microparticle [23,24].

Polymer microcapsules [16,25] are particularly interesting because of their capacity for encapsulating therapeutic agents while avoiding the adverse effect of CaCO_3_ on the cellular calcium balance. They also can be designed to respond to specific stimuli, allowing for targeted drug release within tumors [26,27]. Polymeric microcapsules are synthesized on the basis of CaCO_3_ templates, which are usually dissolved by ethylenediaminetetraacetic acid (EDTA) after LbL assembly of polyelectrolytes [28]. The EDTA concentration determines the dissolution rate and the final properties of the microparticles, including size, porosity, and stability.

Various therapeutic agents, including low-molecular-weight drugs, proteins, and nucleic acids, can be encapsulated by loading into CaCO_3_ cores through absorption or chemical co-precipitation during the formation of the cores [29]. The loading capacity of these systems depends on several factors, such as the porosity and specific surface area of the CaCO_3_ particles and the chemical properties of the drug. Studies have shown significant effectiveness of low-molecular-weight drug encapsulation [30] and their controlled release from CaCO_3_ cores [31], sometimes with a reduced cytotoxicity [32]. The efficiency of encapsulation and stability of encapsulated molecules have been also demonstrated for proteins [33] and nucleic acids [34]. Drug release from delivery systems based on CaCO_3_ microparticles can be activated by external stimuli, such as a change in pH [35] (slightly acidic in tumors) or temperature [22]. For targeted drug delivery, CaCO_3_ microparticles can be functionalized with recognition molecules, usually antibodies, interacting with specific receptors on target cells [36]. Moreover, in vivo studies of a nasal drug delivery system based on CaCO_3_ microparticles has shown improved bioavailability of the active substance [37]. Recently, in vivo applications of CaCO_3_ particles using various administration routes have been intensely studied and proven to be promising [38].

In conclusion, the loading of drugs into calcium carbonate cores, core/shell microparticles based on them, or microcapsules is a promising technique in the field of drug delivery, especially for cancer therapy. CaCO_3_-based microparticles efficiently encapsulate various therapeutic agents, improving their bioavailability and specifically targeting cancer cells while reducing side effects. In this review, we will first discuss the methods of synthesis of calcium carbonate cores and the fabrication of CaCO_3_-based microparticles and microcapsules. Then, we explore the systems for the delivery of small-molecule drugs, proteins, and DNAs based on each of these structures, and finally address the potential uses and key challenges of these microstructures in cancer treatment.

## 2. Core-Only CaCO_3_ Microparticles

Calcium carbonate cores have been used as containers over the past two decades [39] and offer numerous advantages for the delivery of pharmacological compounds, such as biocompatibility, a high loading capacity, and maintenance of the properties of the loaded molecules [40]. Their size and shape vary depending on synthesis conditions, including temperature, reactant concentrations, viscosity of the medium, and reaction time, which allows obtaining cores with desired properties [1,6,7]. The internal porous structure of functionalized calcium carbonate cores is also an important factor influencing drug loading, which has recently been elucidated by mercury intrusion porosimetry and scanning electron microscopy with a focused ion beam [41]. A reduced pore size has been found to be associated with an increased maximum payload, i.e., a higher capacity for retaining compounds within the particles.

### 2.1. Loading Methods

Calcium carbonate cores are used for the loading of small molecules [21,42], proteins [43,44], nucleic acids [34], and radionuclides [45,46]. The substances are loaded into the CaCO_3_ cores either by co-synthesis, when the proteins are captured by the CaCO_3_ cores during their growth, or by the adsorption of loading molecules onto the matrix surface of preformed CaCO_3_ cores [47]. An alternative drug loading method by solvent evaporation is suitable for small molecules with different solubilities [42]. The adsorption of poorly soluble drugs onto the CaCO_3_ particles may help overcome the low bioavailability of drugs [48], whereas loading during co-synthesis leads to the aggregation of proteins [43]. The co-precipitation method has proven to have a high loading efficiency for both small-molecule drugs and proteins [18,49]. The loading efficiency depends on the drug diffusion through the pores at the pH and ionic strength suited to each particular compound, while ensuring the preservation of its bioactivity. For example, the loading of superoxide dismutase into vaterite CaCO_3_ crystals at pH 8.5 was highly efficient, with its activity retained, whereas at pH 9.5, only a 30% retention was achieved [43].

The enhancement of protein encapsulation into 6.9 µm CaCO_3_ microparticles using protein–polysaccharide interactions has been shown [50]. The chitin-binding domain (ChBD) was inserted into a β-lactamase protein (BlaP) to obtain a chimeric protein, BlaPChBD, exhibiting an affinity for hyaluronic acid (HA). In the presence of HA, the particle size was decreased to 4.5 µm, which indicated a templating effect of HA on CaCO_3_. The chitin-binding domain (ChBD) ensured a more stable interaction between the protein and HA, reducing aggregation and decreasing the particle size. The use of supercritical CO_2_ (ScCO_2_) technology in the presence of HA ensured successful encapsulation of BlaPChBD in vaterite CaCO_3_ microparticles, increasing protein encapsulation sixfold compared to BlaP alone. In addition, thrombin cleavage sites were introduced to facilitate protein release by protease cleavage, the release rate being increased from less than 20% to 87% within 36 h. The β-lactamase encapsulation rate was below 1%, apparently due to unfavorable electrostatic interactions at pH 6.5, and was slightly increased (to 1.2%) after the insertion of the chitin-binding domain. The use of HA significantly increased the encapsulation of BlaPChBD (to 6.27%) due to protein–polysaccharide interactions. The results demonstrate the efficacy of using HA for enhancing the encapsulation and controlled release of proteins in CaCO_3_-based delivery systems, offering a promising approach to the development of biodegradable and targeted drug delivery systems.

### 2.2. Demonstration and Limitations

The loading of three therapeutic proteins (insulin, catalase, and aprotinin) into vaterite CaCO_3_ cores has shown that the protein loading capacity is independent of their molecular weight and depends only on inter-protein interactions [44]. The tested proteins differ from one another in adsorption kinetics, which indicates differences in the adsorption mechanisms.

The efficiencies of loading catalase into CaCO_3_ vaterite crystals by means of absorption into preformed crystals (ADS) and co-synthesis (COS) [51] have been compared. COS has been shown to be more efficient, as in the case of the loading of small molecules [18], with a protein content of 20.3% versus 3.5% loaded by the ADS method. The high loading capacity of COS, with a local protein concentration of about 550 mg/mL, was due to CaCl_2_-induced inter-protein interactions resulting in aggregation. The adsorption isotherms better fitted the Langmuir and Brunauer–Emmett–Teller (BET) models than the Freundlich model, which indicated aggregation in solution followed by absorption of aggregates into the crystals. Furthermore, catalase was found to retain about 79% of its specific activity after ADS loading. The stability of the aggregates in the crystals was confirmed by the fact that catalase loaded by the COS method could not be effectively removed by a single washing, unlike catalase loaded by the ADS method. This study highlights the high potential of the COS method for loading large amounts of active proteins into CaCO_3_ crystals, offering a new approach to the encapsulation of therapeutic proteins [51]. One of the main problems with vaterite CaCO_3_ particles is their aggregation [25]. However, stabilizers, such as SDS, successfully overcome this problem [21].

**Figure 2:**
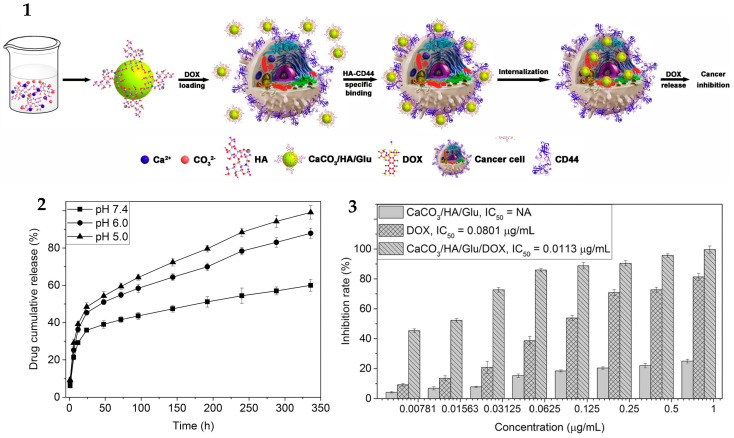
(**1**) Preparation of CaCO_3_/HA/Glu MHSs, efficient loading of DOX, targeted delivery, specific internalization, and significant inhibition of cancer cells. (**2**) In vitro release profiles of CaCO_3_/HA/Glu/DOX under different pH. Data represent the mean ± S.D.; n = 3. (**3**) Cytotoxic effects of free DOX, CaCO3/HA/Glu, and CaCO^3^/HA/Glu/DOX on HeLa cells after 3 d treatment. Data represent the mean ± S.D.; n = 3. Abbreviations: HA, hyaluronate; Glu, glutamate; MHSs, mesoporous hollow spheres; DOX, doxorubicin. Adapted with permission from Guo, Y. et al., J. Coll. Interf. Sci.; published by Elsevier, 2017 [52].

The CaCO_3_-based delivery systems are often designed to be pH-dependent. Calcium carbonate/hyaluronate/glutamate submicron hollow spheres loaded with doxorubicin (DOX) [52] released 59.97% of DOX within 14 days at pH 7.4, 87.89% at pH 6.0, and 99.15% at pH 5.0, with a loading efficiency of 85%. Specific binding of these particles to cancer cells was provided by the ligand–receptor interaction between HA and CD44 receptors, overexpressed on cancer cells. The IC_50_ of DOX-loaded microspheres was much lower than that of free DOX when tested on HeLa cancer cells (Figure 2).

Pneumolysin (PLY)-loaded CaCO_3_ particles (0.95 µm) containing ovalbumin as a model antigen have been developed as a multimodal antigen delivery system for antitumor vaccines. OVA/CaCO_3_/PLY nanoparticles obtained by physical adsorption of OVA and PLY on CaCO_3_ promoted lysosomal degradation, cytoplasmic release, and cross-presentation of antigens, enhancing cellular immunity. The OVA/CaCO_3_/PLY system induced efficient lysosomal leakage and cytoplasmic delivery of OVA in vitro [53].

The kinetics of drug release from the systems based on CaCO_3_ cores is often bimodal, with initially rapid release due to the dissolution of aggregates followed by sustained release [54]. As the vaterite crystals destabilize, their morphology changes into the calcite one, making the release irreversible. The presence of aggregates within the matrix and the high loading rate by the co-synthesis method, especially for proteins, indicate the limitations of the application of the loading method by adsorption [51]. Nevertheless, other CaCO_3_-based particle systems are being developed and exhibit a high efficiency in substance delivery. Table 1 summarizes the characteristics of the systems based on CaCO_3_ cores as vehicles for the delivery of small molecules, proteins, DNAs, and radionuclides.

## 3. CaCO_3_-Based Core/Shell Systems

### 3.1. Methods of Fabrication

The CaCO_3_ particles coated with a polyelectrolyte shell are better suited for the delivery of drugs and proteins. Polyelectrolytes are deposited onto the cores by the LbL method [63,84] or by electrospray [85]. Variation of the number of cationic/anionic bilayers deposited on the particle surface allows better control of the kinetics of substance delivery to the target. Application of these polymers is driven by electrostatic interaction, through covalent or hydrogen bonds, which explains how the release of loaded molecules can be induced by different stimuli, such as pH, ionic strength, temperature change, or ultrasound.

**Figure 3:**
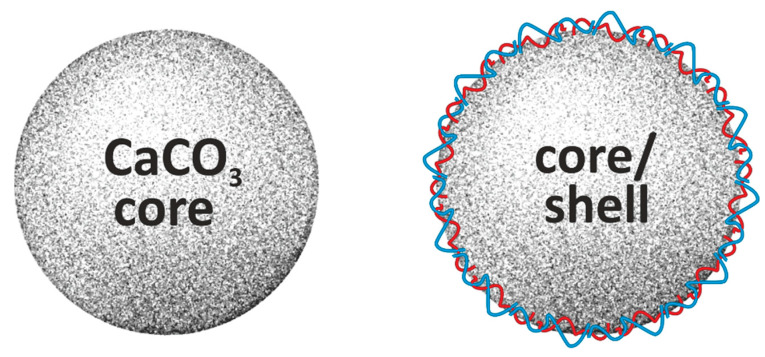
Summary of data on fabrication of porous CaCO_3_ core-only and CaCO_3_ core/shell microparticles. **On the left:** the pore size diameters for differently fabricated CaCO_3_ cores are shown to be in the range of 2–50 nm [21], 5–30 nm [43], 10–60 nm [44] or 20–500 nm [49]. **On the right:** the shells on the CaCO_3_ cores may be fabricated by the deposition of different polymers such as poly-L-ornithine/fucoidan [56]; poly(ethylene glycol)/oleic acid [57]; hyaluronic acid/glutamate [52]; hyaluronic acid/tannic acid [60]; ovalbumin/platelet lysate [53]; poly(diallyldimethylammonium chloride)/poly(sodium 4-styrenesulfonate) [58]; hyaluronic acid [50]; polylactic acid [61]; poly(acrylic acid) [62].

For the encapsulation of therapeutic agents, adsorption or co-synthesis can be used, and the choice of method determines their location: on the surface, between the layers, or within the matrix. Figure 3 summarizes the data on the fabrication of CaCO_3_ core-only and core/shell microparticles.

### 3.2. Delivery of Small Molecules

**Figure 4:**
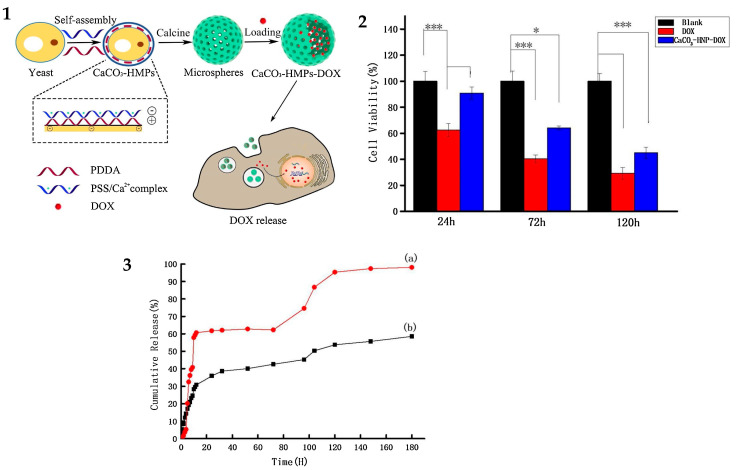
(**1**) Assembly schematic: the preparation of CaCO_3_-HMPs through self-assembly of two oppositely charged polyelectrolytes, PDDA and PSS, on the surface of yeast cells, as dual templates for drug loading and release. (**2**) Cytotoxicity tests of CaCO_3_-HMPs, DOX, and the CaCO_3_-HMPs-DOX drug-delivery system (* *p* < 0.05, *** *p* < 0.001); (**3**) Cumulative release curve of DOX in different environments: (a) pH = 4.8 and (b) pH = 7. Abbreviations: HMPs, herbal medicinal products; PDDA, poly(diallyldimethylammonium chloride); PSS, poly(sodium 4-styrenesulfonate); DOX, doxorubicin. Reproduced with permission from Wei, Y., et al. Coll. Surf. B Biointerf.; published by Elsevier, 2021 [58].

In the development of systems for small-molecule delivery based on microparticles, DOX is often used as a model anticancer drug. Efficient loading of DOX has been shown for core/shell microparticles composed of ∼2 µm CaCO_3_ cores coated with poly-L-ornithine and fucoidan. The release of DOX from these particles was confirmed by a significant antiproliferative effect on MCF-7 breast cancer cells [56]. DOX-loaded CaCO_3_ microparticles modified with oleic acid (OA) and polyethylene glycol (PEG) exhibited a 70% drug release within 2 h in cancer cells in response to their specific environment, whereas their stability and drug retention in various other aqueous media were enhanced. Hybrid CaCO_3_ microspheres have also been obtained using yeast cells as the organic matrix and the polyelectrolytes poly(diallyldimethylammonium chloride) (PDDA) and sodium poly(styrene sulfonate) (PSS) as shell components, with subsequent calcination and DOX loading [58]. Drug release tests showed an accelerated release of DOX in an acidic environment (pH 4.8) typical of cancer tissues compared with a neutral medium (pH 7). Cytotoxicity tests have shown a good biocompatibility of CaCO_3_ microparticles 3 µm in diameter loaded with herbal medicinal products (HMPs) (Figure 4). Gradual decomposition of the coated particles in the acidic microenvironment of tumors ensures the targeted release of the drug directly into the cancer cells, thereby improving the efficacy of the treatment and minimizing the side effects on the surrounding healthy tissue. Thus, the feasibility of the delivery of small molecules using the core/shell system has been demonstrated.

### 3.3. Delivery of Proteins

Calcium carbonate microparticles containing cancer cell lysate and coated with polymer substituted with the low-molecular-weight TLR7/8 agonist have been developed, which could serve as novel personalized anticancer vaccines [62].

The solid-in-oil-in-water emulsion method for the manufacture of CaCO_3_/polylactic acid core/shell microparticles about 1.11 µm in size has been designed as a tool for the controlled transport and release of water-soluble bioactive molecules. This technology could be used for developing more effective drug delivery systems [61].

The biomimetic approach has been used to obtain core/shell microparticles with a liquid core consisting of charged emulsion droplets or liposomes and a CaCO_3_ shell, which can also be used as delivery vehicles [62].

Overall, these techniques improve the encapsulation and release of proteins, offering promising advances for drug delivery systems.

### 3.4. Delivery of Nucleic Acids

Although encapsulation of nucleic acids in core/shell systems has not yet been reported, some studies envisage it. For example, Bewernitz et al. [62] explore the manufacture of liquid-core/solid-shell microcapsules representing CaCO_3_-coated emulsions and liposomes. These microcapsules, ranging in size from 2 to 10 µm, have been designed for potential applications in the controlled release of substances, including DNA molecules. The method relies on the precipitation of CaCO_3_ to form a shell around emulsion droplets or liposomes. This approach could be used to engineer a promising system for the protection and targeted delivery and release of DNA in biomedical applications due to the possibility of controlling the permeability and degradation of the CaCO_3_ shell.

Applications of CaCO_3_ core-based core/shell microparticles are summarized in Table 1.

## 4. CaCO_3_-Based Hollow Microcapsules

### 4.1. Methods of Fabrication

Calcium carbonate-based hollow (or shell) microcapsules represent a fascinating area of research in medical nanotechnology, providing unique opportunities for targeted cancer treatment [29]. These microcapsules with encapsulated therapeutic agents are often designed to interact directly with tumors by functionalization of their surface with antibodies, peptides, proteins, hyaluronic acid, or nucleic acids to ensure controlled, targeted drug delivery [86].

**Figure 5:**
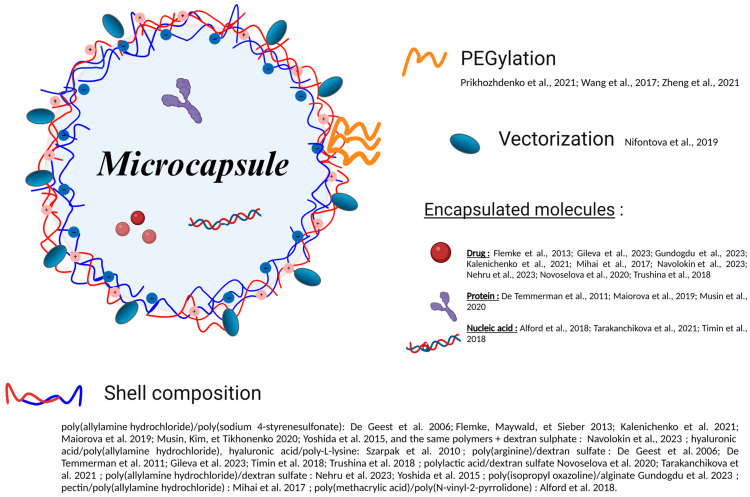
Summary of data on fabrication of microcapsules initially based on the CaCO_3_ microparticles [18,20,22,29,32,36,57,64,65,67,68,69,70,72,73,75,76,77,78,79,81,89].

The fabrication of these microcarriers is based on the LbL assembly of polyelectrolytes, a technique first tested on metformin particles [24], which allows the construction of multilayer films with nanometric precision by alternating the immersion of a substrate in solutions of polyelectrolytes of opposite charges. CaCO_3_ cores, whose synthesis was considered above, are used as templates for the fabrication of microcapsules. Then, the cores are dissolved with a chelating agent, e.g., EDTA, and washed, and hollow spherical polyelectrolyte capsules are thus formed. Polyelectrolytes in different combinations, such as poly(allylamine hydrochloride) (PAH) and PSS [18,87], PAH and poly(vinyl sulfate) (PVS) [20], chitosan (Chi) and alginate (Alg) [82], HA and PAH/poly-L-lysine (PLL) [64], and poly-L-arginine (pArg) and dextran sulfate (DS) [65], are particularly effective in forming these multilayers on vaterite CaCO_3_ cores. Detailed comparison of the stabilities, shrinkabilities, and internal structures of capsules made of different biopolymers have been performed [15]. These polymers, selected for their capability for self-assembling, ensure high stability and functionality of the microcapsules, making it possible to modulate their properties, such as solubility, reactivity, and biological compatibility, for the purposes of biomedical engineering and formation of protective coatings and sensors [88]. Figure 5 summarizes the methods for obtaining shell microcapsules.

### 4.2. Delivery of Small Molecules

The capability of multilayered polyelectrolyte capsules to host low-molecular-weight drugs for cancer targeting has been recently demonstrated [30]. These smart polymer capsules exhibit considerable versatility, paving the way for future developments in medical nanotechnology and personalized medicine [66]. In recent years, uniformly sized microcapsules obtained on the basis of CaCO_3_ cores as removable templates, have been loaded with gemcitabine and clodronate [70], DOX [18], apigenin and ascorbic acid [69], curcumin and ciprofloxacin [22], and *Gratiola officinalis* extract [68] as model drugs for cancer and other diseases.

Different encapsulation approaches are used with small-molecule drugs. Microcapsules fabricated using the PAH and PSS polyelectrolytes on CaCO_3_ cores have exhibited efficiencies of DOX loading by co-precipitation and spontaneous loading of about 73 and 65%, respectively, due to optimized pH and salt concentration [18]. PAH/dextran sulfate (DS) polymer microcapsules designed for the delivery of apigenin and ascorbic acid exhibited a loading efficiency of about 20% for each substance after incubation of the microcapsules in the presence of the drugs [69]. The gemcitabine loading efficiency of submicron pArg/DS microcapsules was about 47% [70].

The microcapsules are designed so as to release the loaded drugs in response to specific stimuli. In the case of PAH/DS capsules containing apigenin and ascorbic acid, in vitro release was 45% and 40%, respectively, after 2 h at the physiological pH [69]. This study has also shown that the chemical composition of the capsules strongly affects the drug solubility and rate of its release. The release of DOX by diffusion from PAH/PSS microcapsules was prolonged at pH 6.0 and 7.4, corresponding to the pH values in tumor and normal tissues, respectively. The cumulative release of DOX within 48 h did not exceed 70% [18].

**Figure 6:**
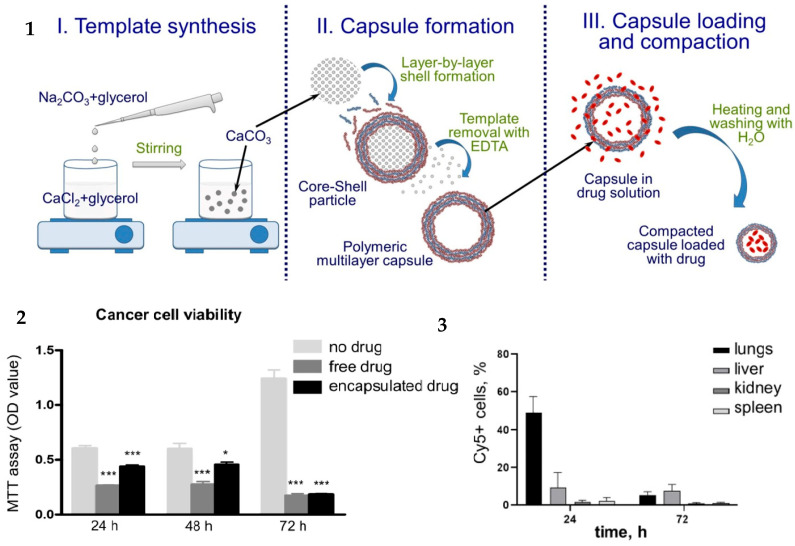
(**1**) Scheme of the stepwise capsule assembly, compaction, and loading. (**2**) Lung cancer cell viability in the presence of 20 μM free or encapsulated gemcitabine; MTT assay at the indicated time points. (**3**) Number of cells in the lungs, liver, kidney, and spleen with internalized Cy5-labeled capsules relative to the total amount of cells in the respective organs 24 and 72 h after intravenous injection of PMC. Abbreviation: PMC, polymeric multilayer capsules. Adapted with permission from Novoselova, M. V., et al. ACS Appl. Mater. Interfaces; published by American Chemical Society, 2020 [70].

In in vitro experiments, pArg/DS microcapsules loaded with gemcitabine were internalized at a rate higher than 75% by macrophages and lung and liver epithelial cells [70]. Experiments in mouse models showed the specificity of microcapsule delivery: they were better retained by lung tumor than by healthy lung tissue. The efficiency of encapsulated gemcitabine estimated by the MTT assay was lower than that of the free drug after 24 and 48 h of incubation and equal to it after 72 h of incubation, which confirmed the prolonged, gradual release of the drug (Figure 6).

Microcapsules are commonly developed to reduce the side effects of drugs and to allow a more prolonged and targeted action of, e.g., DOX, thereby improving the efficacy of the treatment. It can be co-administered, thus compensating for the rapidity of elimination from the body [32]. Also, the microcapsules containing *Gratiola officinalis* extract were shown to effectively release the drug, causing the death of 100% of cultured cancer cells through overcoming protective autophagy [68].

Hollow polymeric microcapsules are also used for the encapsulation of live *E. coli* cells. CaCO_3_ cores containing *E. coli* cells were obtained by co-precipitation and coated with different polyelectrolytes. Then, CaCO_3_ cores were dissolved in EDTA to obtain capsules with a size of about 5 μm. Encapsulation reduced cell viability, the effect being mainly accounted for PAH, with only minor contributions from the other components. The encapsulated cells exhibited a prolonged lag phase of growth while retaining the ability to produce green fluorescent protein. About 40% of cells were alive after the encapsulation. This method has potential applications in the high-throughput screening of biocatalyst libraries, requiring optimization to improve cell survival [73].

Composite microcapsules based on CaCO_3_ have been developed that contain various types of pectin with different degrees of methylation and amide content, as well as mixtures of polyelectrolyte complexes, including poly(allylamine) hydrochloride. These CaCO_3_/pectin capsules were used as matrices for the loading of tetracycline hydrochloride (TCH), with analysis of drug release kinetics using the Higuchi and Korsmeyer–Peppas models. In vitro assays demonstrated the influence of CaCO_3_ polymorphs on the drug release process, with 22–27% of TCH released within 10 h at pH 7.4 [72].

The potential of using CaCO_3_-templated PAH/PSS polymer capsules for the targeted delivery of vitamin B12 has also been demonstrated [75]. The successful encapsulation of vitamin B12 was confirmed by optical absorption spectroscopy, transmission electron microscopy, and atomic force microscopy data. Experimental data on the specific encapsulation capacity of these polymer capsules for vitamin B12 show their potential as targeted vectors for nutrient delivery, highlighting the effectiveness of the PAH/PSS system in developing biocompatible and stable drug-delivery vectors.

### 4.3. Delivery of Proteins

Proteins can also be transported and released by polyelectrolyte capsule systems assembled on CaCO_3_ cores [90]. The chemical methods for the fabrication and post-modification of hollow polymer capsules for protein delivery, including covalent bonding, electrostatic attachment, and hydrogen bonding, have been described [91]. Proteins can be encapsulated by physical adsorption on preformed CaCO_3_ cores or by co-precipitation during the CaCO_3_ particle synthesis. The latter approach has been shown to be five times more efficient [71]. Horseradish peroxidase (HRP) and ovalbumin serving as model antigens have been encapsulated in CaCO_3_-based pArg/DS polymer capsules by co-precipitation. After lyophilization in the presence of polyols, HRP retained up to 70% of its enzymatic activity. Ovalbumin-loaded microcapsules were used as a model vaccine formulation. Ovalbumin encapsulated in polyelectrolyte microcapsules caused enhanced antigen presentation and amplification of T-cell proliferation compared to soluble ovalbumin. The immunological activity of lyophilized microcapsules was preserved according to the results of in vitro T-cell proliferation assay [77].

The effect of pH on the degradation of polyelectrolyte microcapsules formed on CaCO_3_ particles with proteins encapsulated by adsorption was also studied [76]. An increase in pH led to an increase in protein yield and PAH detachment, apparently because the acidity of the medium (pH 7) was close to the charge exchange point of the PAH amino group. A high concentration of NaCl (2 M) caused considerable PAH dissociation and release of the protein.

### 4.4. Delivery of Nucleic Acids

Studies using polymeric capsules for delivering genetic material into cells are also carried out. CaCO_3_-based microcapsules made from biodegradable biopolymers were used for the delivery of all CRISPR-Cas9 components to cells [79]. The efficiency of transfection indicated by a loss of red fluorescence in dTomato-expressing HEK293T reached 70%. Submicro- and microcapsules with pArg/DS shells were successfully used as carriers for messenger RNA (mRNA) and small interfering RNA (siRNA) [78]. This study demonstrated that the package efficiency of RNA molecules, delivery efficiency, and biodistribution strongly depended on the size of the capsules. Both studies highlight the importance of developing safe and effective delivery systems for gene therapy and genome editing. The use of microcarriers offers a promising alternative to viral vectors, reducing the associated risks and potentially enhancing the clinical acceptance of these technologies. The delivery systems based on microcapsules are summarized in Table 1.

Finally, the use of CaCO_3_-based microcapsules in various medical applications, especially in immunotherapy and targeted cancer treatment, appears a promising approach. Ongoing research and innovations in this field could transform cancer treatment, offering more effective and less invasive solutions, notably through the release of small molecules, proteins, and nucleic acids encapsulated in these polyelectrolyte capsules by physical absorption or co-precipitation, thus marking a significant evolution in therapeutic strategies.

## 5. Conclusions

CaCO_3_ microparticles are promising tools for anticancer therapy for several reasons: they are capable of incorporating a wide spectrum of active substances, both low-molecular-weight ones and biological macromolecules, and their size and pH sensitivity can be varied, which is advantageous for the controlled delivery of drugs, including gene therapy agents. Of special importance for targeted cancer therapy is the enhanced permeability and retention (EPR) effect of CaCO_3_ microparticles ensuring their ready penetration into tumors and subsequent degradation and release of the loaded agent in the acidic microenvironment characteristic of malignant tumors [92,93]. CaCO_3_ particles have been shown to cause the reprogramming of cancer cells and inhibition of tumor growth [5].

CaCO_3_ submicro- and microparticles have considerable potential as vectors for targeted drug delivery, particularly in cancer treatment. Their controlled dissolution depending on pH ensures targeted drug release in the acidic areas of tumors while maintaining stability in the more neutral circulatory system. Different configurations of the delivery system, core-only and core/shell microparticles and microcapsules, offer solutions for the transport and controlled release of various therapeutic substances, including small molecules, proteins, and nucleic acids. Other materials have been studied in this respect, including metformin, manganese carbonate (MnCO_3_), and cadmium carbonate (CdCO_3_). However, metformin is insufficiently biocompatible, and both MnCO_3_ and CdCO_3_ microparticles are considerably smoother than CaCO_3_ ones and may be toxic. In contrast, CaCO_3_ microparticles are highly biocompatible, the roughness and porosity of their surface enabling adhesion of polymer layers to form a thick shell. For example, a (PAH/PSS)_4_ is about two times thicker than the shells that can be deposited onto MnCO_3_ and CdCO_3_ cores, with polymer layers formed not only over the particle, but also on the inner surface of the pores [63].

Vaterite CaCO_3_ cores are effective for loading small molecules through techniques such as co-precipitation, allowing for their subsequent controlled release. However, their rapid degradation in vivo can lead to premature release and disrupt the cellular calcium balance. To address this issue, core/shell particles have been developed, where the CaCO_3_ core is coated with a shell of polyelectrolytes, which regulates its degradation, thus allowing sustained and controlled drug release while minimizing cell damage. This system can also be modified to specifically target cells or tissues, improving therapeutic efficacy and reducing side effects.

Finally, CaCO_3_-based polyelectrolyte capsules overcome the issues entailed with CaCO_3_ particles. Removal of the core through calcium chelation limits the destabilization of the tumor microenvironment by the increase in intracellular Ca^2+^ and ultimately controlling the pH. The capsules are particularly promising for the encapsulation and controlled release of small molecules, nucleic acids, and proteins, due to their ability to degrade under specific intracellular conditions. Although the delivery of biomacromolecular therapeutic agents presents a huge challenge compared to the delivery of small molecules due to both their high molecular weight and fragile structure, these problems can be solved by using polymer delivery systems [94]. In summary, CaCO_3_-based particles offer a versatile platform for more effective therapeutic treatments, particularly for complex diseases, such as cancer, due to their adaptability and capability for targeted and controlled drug delivery and release.

## 6. Outlook: In Vivo Studies

### 6.1. Modulation of the pH of Tumor Environment

Submicron CaCO_3_ particles offer a promising tool to counteract the characteristic acidity of tumors, a known factor in promoting their aggressiveness and metastatic potential. The targeting of tumors with 20 to 300 nm calcium carbonate particles allows for the gradual increase of the tumor pH to neutrality. This pH modulation is crucial, because a less acidic environment can inhibit the growth and spread of cancer cells, thereby reducing their virulence. Particularly, 100 nm particles stand out for their ability to sustain a prolonged pH elevation. This highlights the importance of particle size optimization in maximizing the treatment efficacy. Tests on animal models have shown a significant reduction of tumor growth, attesting to the therapeutic potential of this method. However, further research is required to optimize the dosage, evaluate the synergy with other treatments, and predict side effects. This advancement shows a way for improving cancer treatment strategies by targeting a fundamental aspect of tumor biology [95].

### 6.2. Biodistribution and Biocompatibility

The in vivo biodistribution of capsules is a major issue for the development of safe and effective drug carriers. Fluorescent CaCO_3_-based pArg/DS capsules have been developed for kidney targeting via the renal artery [81]. The high efficiency of delivery to the area of interest was provided by the optimization of the administration protocol and dosage.

### 6.3. Retention, Stability, and Toxicity

CaCO_3_ particles labeled with ^224^Ra were proposed for the local therapy of disseminated tumors using intraperitoneal administration [46]. The results showed a targeted localization of microparticles, with moderate systemic payload release. Biodistribution studies showed that radioactivity was primarily localized in the peritoneal area after administration, with the highest activity associated with intraperitoneal adipose tissue and the parietal peritoneum. The release of ^224^Ra from the particles was relatively limited, as evidenced by reduced absorption in the skeleton compared to the administration of free ^224^Ra. Non-abdominal organs, such as the heart, muscles, and brain, displayed radioactivity levels below 100 Bq/g, which indicated a limited radiation exposure outside the abdominal area. These results indicate that radiolabeled CaCO_3_ particles possess a high retention capacity and targeted bioavailability, making them potentially useful for targeted medical applications, minimizing non-target tissue exposure to radiation. The antitumor effect of CaCO_3_ microparticles labeled with the alpha-emitting ^224^Ra was shown in mice [45]. This study highlights the advantage of using CaCO_3_ as a carrier of therapeutic agents and shows a particularly promising therapeutic strategy for tumors located in the abdominal cavity.

CaCO_3_ core/shell particles 0.8 µm in size were used for the encapsulation of the alpha-emitting ^225^Ac in order to enhance its retention and reduce systemic toxicity during alpha therapy [60]. The study showed a 93–94% retention of ^225^Ac after 20 days, with the majority of ^225^Ac microparticles localized in the lungs, which indicated a reduced renal toxicity potential. In vivo tests on Wistar rats confirmed the high retention efficiency of the particles, underscoring the effectiveness of ^225^Ac-doped core/shell particles in safely retaining alpha emitters used for cancer treatment.

The wide potential applications of CaCO_3_ nanoparticles in various sectors, including medicine, calls for a thorough evaluation of their toxicity. In vitro experiments on NIH 3T3 and MCF7 cells treated with CaCO_3_ nanoparticles at different concentrations (1–50 μg/mL) for 12 to 72 h showed no cytotoxicity, oxidative stress, or DNA damage, indicating excellent biocompatibility. In vivo studies with zebrafish treated with CaCO_3_ nanoparticles at doses as high as 200 μg/mL showed an absence of significant toxic effects on embryonic development. These results underscore the safety of CaCO_3_ nanoparticles, suggesting their applicability in medicine and other fields, without cytotoxic or genotoxic risks to biological systems [96].

Various administration routes have been used for submicrometer- and micrometer-sized CaCO_3_ microparticles, including intranasal [97,98], inhalatory [99], and transdermal [100] ones. The efficiency of drug delivery to the brain by CaCO_3_ carriers administered intranasally has been demonstrated by in vivo functional tests [97]. Submicron (0.65 µm) CaCO_3_-based particles administered by inhalation exhibited the highest efficiency of delivery to the blood and the respiratory part of the lung [99]. The transdermal administration of CaCO_3_-based particles has also been tested. Prolonged drug release and the possibility of both targeted and systemic delivery have been demonstrated [100].

### 6.4. Vaccinal Applications

CaCO_3_ microparticles are better than currently used vaccine delivery vehicles, including liposomes, synthetic copolymer systems, and metal nanoparticles, in several respects. Applications of liposomes for vaccine delivery are being developed, some of them being already available. However, their use is seriously limited because liposomes are prone to aggregation, premature vaccine release, and collapse under the conditions encountered in vivo. They sometimes poorly penetrate through biological membranes, and liposomes consisting of positively charged lipids may be toxic [101,102].

In addition, the production of liposome drug delivery systems is much more expensive than the production of conventional drugs: the production cost of 1 g of CaCO_3_ is estimated to be as small as $0.2–0.4, whereas the production of 1 g of liposomes costs over $100 [103]. Therefore, alternative vaccine delivery tools are needed. Mineral microparticles, in particular, calcium carbonate beads, are more stable, biocompatible, and/or biodegradable and are less expensive to produce [103].

Recent studies illustrate the innovative use of vaccines in anticancer immunotherapy, highlighting the in vivo efficacy of formulations based on submicron- and micron-sized CaCO_3_ particles. The physical adsorption of an antigen (ovalbumin) into CaCO_3_ particles with adsorbed pneumolysin, the key virulence factor of *Streptococcus pneumoniae*, significantly amplified cellular and humoral immunity, demonstrating preventive and therapeutic antitumor efficacy [53]. The 0.95 µm CaCO_3_ particles degraded into Ca^2+^ and CO_2_ in the acidic lysosomal environment, promoting cross-presentation of antigens. This biodegradability of the particles was confirmed by the detection of intracellular Ca^2+^, with the highest levels observed for the ovalbumin/CaCO_3_/pneumolysin group. This study illustrates the induction of a robust immune response, offering an effective platform based on submicron- and micron-sized CaCO_3_ particles for the development of anticancer immunotherapy through vaccination.

The last but not the least, it is well known that the translation from experiments to clinical trials is hindered not only by high production costs and the problems with scale-up, but also by safety issues and complicated procedures of obtaining approval by drug administration authorities. However, this problem does not exist in the case of calcium carbonate microparticles because their safety is guaranteed by the fact that they are already marketed as an FDA-approved antacid medication, as well as a digestive, antidiarrheal, and weight control drug [104].

## Figures and Tables

**Table 1:** The key characteristics and results obtained with the CaCO_3_ core-only and CaCO_3_-based core/shell microparticles and shell-only microcapsules.

Particle Type	Size	Cargo Type	Encapsulated Molecule	Results/Conclusions of the Cited Study	Shell Composition	Ref.
Core	1 μm	Small molecule	Doxorubicin	The IC50 value of doxorubicin for HeLa cancer cells is 0.0113 μg/mL, much lower than that of the free doxorubicin (0.0801 μg/mL).	Hyaluronate/glutamate	[52]
Core	0.6–3.2 µm	-	-	The thermal expansion of vaterite is reported for the first time; vaterite is not converted to calcite in the dilatometric test.	-	[6]
Core	0.43 µm	Fluorescent dye	Rhodamine 6G	Submicron vaterite containers enable controlled loading and release of active substances via calcite recrystallization in water.	-	[40]
Core	0.52 µm	-	-	The optimal salt concentrations, reaction time, and organic additives for ensuring controllable and reliable CaCO_3_ particle design have been estimated.	-	[1]
Core	0.4–2.7 µm	-	-	Submicron vaterite particles have been synthesized by the drop precipitation method from saturated solutions of NaCO_3_ and CaCl_2_ in the presence of ethylene glycol with an EG-to-H_2_0 ratio of 4:1.	-	[7]
Core	n/a	Protein	BSA	Imaging the internal structure of functionalized CaCO_3_ using FIB-SEM combined with MIP has distinguished between open, blocked, and drug-laden pores.	-	[41]
Core	17.9 μm	Small molecule	Ibuprofen, nifedipine, losartan potassium, and metronidazole benzoate	Drug loading by solvent evaporation is simple, fast, and efficient.	-	[48]
Core	3.1–23.5 µm	Small molecule	Aspirin, vanillin	The mechanism of adsorption of active ingredients onto functionalized CaCO_3_ particles has been discovered, which could enable tailored loading without altering the efficacy of the ingredients when released.	-	[42]
Core	17.9 μm	Protein	Lysozyme, BSA	Functionalized CaCO_3_ particles are a suitable pharmaceutical excipient for the delivery of proteins, such as lysozyme, with a loading efficiency of over 90%.	-	[49]
Core	3.4 μm	Protein	Superoxide dismutase	Superoxide dismutase (SOD) can be efficiently loaded into vaterite CaCO_3_ crystals, with a content in the crystals as high as 380 mg/mL (10^–2^ M). SOD co-synthesis at pH 8.5 fully preserves SOD bioactivity.	-	[43]
Core	10 μm	Protein	Catalase, insulin, aprotinin	The loading of three therapeutic proteins (250 kDa catalase, 5.8 kDa insulin, and 6.5 kDa aprotinin) into crystals of different porosities have shown that protein loading capacity depends solely on inter-protein interactions in the bulk solution in the presence of crystals and within the crystals.	-	[44]
Core	0.8–1.6 μm	Small molecule	Doxorubicin	Vaterite CaCO_3_ crystals release drugs, the initial release as low as <10% within 24 h at pH 6. These vaterites exhibit prolonged drug release: ~40% over 8 days at pH 6.	-	[21]
Core	4–5 μm	Protein	Catalase	Extremely large capacity of loading by co-synthesis (~550 mg/mL) is explained by intermolecular protein interactions, i.e., formation of protein aggregates induced by CaCl_2_ during co-synthesis.	-	[51]
Core	1 μm	Protein	Ovalbumin, pneumolysin	Multimodal delivery systems for CaCO_3_/PLY antigens have been developed. OVA/CaCO_3_/PLY vaccine formulations promote antigen cross-presentation, boost cellular and humoral immune responses, and offer promising preventive and therapeutic antitumor efficacy.	-	[53]
Core	5.45 μm	Protein	β-lactamase	β-Lactamase can associate with hyaluronic acid and be successfully loaded into vaterite CaCO_3_ microparticles using the supercritical CO_2_ technology aided by the templating effect of hyaluronic acid on CaCO_3_.	-	[50]
Core	1.3 μm	-	-	Intravenous injection of CaCO_3_ particles at a dose of 50 mg/kg significantly disrupted red blood cells but did not induce visible abnormalities in the tissue structures of key organs.	-	[55]
Core	4–7 μm	Radionuclide	^224^Ra	^224^Ra-labeled CaCO_3_ microparticles are a promising agent for therapy against cancer dissemination in body cavities. A significant therapeutic effect has been obtained at specific activities from 0.4 to 4.6 kBq/mg.	-	[45]
Core	1–3, 3–15 μm	Radionuclide	^224^Ra	^224^Ra-labeled CaCO_3_ microparticles remain in the peritoneal cavity, with a modest distribution of ^224^Ra systemically, when administered at a relevant microparticle dose.	-	[46]
Core	0.2–1.1 μm	Nucleic acid	DNA	CaCO_3_/calcium phosphate (CaP)/DNA nanoparticles have a reduced size, better stability, and significantly higher gene transfection efficiency compared to CaCO_3_/DNA and CaP/DNA ones.	-	[34]
Core/shell	2 μm	Small molecule	Doxorubicin	Doxorubicin has been loaded into CaCO_3_ microparticles coated with poly-L-ornithine/fucoidan with a loading efficiency as high as 69.7%. Controlled release of doxorubicin significantly inhibits the proliferation of breast cancer cells.	Poly-L-ornithine/fucoidan	[56]
Core/shell	0.2 μm	Small molecule	Doxorubicin	PEG/oleic acid–amorphous calcium carbonate improves the stability of CaCO_3_-core microparticles in aqueous media and controls drug release in cancer cells, thereby achieving an anticancer efficacy comparable to that of free drugs.	Oleic acid/PEG	[57]
Core/shell	3 μm	Small molecule	Doxorubicin	CaCO_3_ microparticles loaded with herbal medicinal products exhibit excellent biocompatibility and pH sensitivity, which demonstrates their potential as effective drug carriers.	PDDA/PSS	[58]
Core/shell	∼10 μm	Protein	Ovalbumin, cancer cell lysate	CaCO_3_ microparticles are capable of delivering vaccines to cancer cell lysates and exhibit lower cytotoxicity and greatly enhanced cellular uptake leading to improved cross-presentation efficiency.	Poly(HPMA-APMA) with TLR7/8 agonists	[59]
Core/shell	0.65, 3.2 μm	Radionuclide	^225^Ac	CaCO_3_ core-shell particles effectively retain large amounts of ^225^Ac and its daughter isotopes (^221^Fr and ^213^Bi). The kidney accumulation of ^213^Bi after administration of ^225^Ac encapsulated in CaCO_3_ core/shell particles was low, unlike with non-encapsulated ^225^Ac.	HSA/TA	[60]
Core/shell	∼2 μm	Protein	BSA	A CaCO_3_-to-pneumolysin (PLA) mass ratio of 0.8 is optimal in terms of a large protein payload of the microparticles and their stability against dissolution. Bioactive cargos remain intact in pores of PLA-coated CaCO_3_ microparticles.	PLA	[61]
Core/shell	2–4 μm	Fluorescent dye	Nile Red, rhodamine 110	Oil-in-water emulsion droplets and phospholipid bilayer liposomes have been coated with CaCO_3_ to obtain core/shell particles accommodating both hydrophobic and hydrophilic active agents.	CaCO_3_	[62]
Core/shell, shell	2–2.5 μm	Small molecule	Doxorubicin	Doxorubicin can be effectively incorporated into CaCO_3_ microbeads via co-precipitation during their synthesis and into polymer microcapsules via spontaneous loading in an alkaline medium with a Cl^–^ counterion.	PAH/PSS/QD	[18]
Shell	4.75 μm	Protein	Lactalbumine, lysozyme, horseradish peroxidase, chymotrypsin	A new method of protein encapsulation by adsorption into microcapsules obtained through layer-by-layer deposition onto CaCO_3_ cores has been proposed.	-	[39]
Shell	5.4 μm	-	-	Calcium, cadmium, and manganese carbonate crystals have been used as core materials to fabricate hollow polyelectrolyte capsules using layer-by-layer assembly.	PAH/PSS	[63]
Shell	9 μm	-	-	The structure–property relationships have been evaluated for 16 types of capsules made of different biopolymers and the mechanism of capsule formation has been inferred.	PLL, PR, DA, COL/HA, CS, DS, HS	[15]
Shell	3–6 μm	Protein	Insulin	Insulin is released from the microcapsules faster at pH 9.0 and 7.4 than in acidic solutions due to the difference in PAH charge density.	PAH/PSS, PVS, DS	[20]
Shell	5 μm	Fluorescent dye	FITC-dextran	Hyaluronic acid (HA)/poly(L-lysine (PLL) and HA/poly(allylamine) (PAH) capsules are rapidly internalized into endo-/lysosomatic vesicles upon addition to a macrophage culture.	HA/PAH, PLL	[64]
Shell	3 μm	Fluorescent dye	FITC-dextran	Polyelectrolyte capsules containing an enzymatically or hydrolytically degradable polycation degrade spontaneously in VERO-1 cells.	pARG/DS, p(HPMA-DMAE)/PSS, PAH/PSS	[65]
Shell	∼1 μm	Fluorescent dye, protein	Rhodamine B, methylene blue, insulin	Polysaccharide-based glucose-receptive capsules have been made reactive to glucose by attaching a phenylboronic moiety to alginate.	Phenylboronic –modified alginate/PVPON	[66]
Shell	1.8–3.8 μm	-	-	Polyarginine/dextran sulfate (pArg/DS) capsules shrink and densify when heated, with their thermal response unaffected by the initial size, number of layers, or layer sequence.	pArg/DS	[67]
Shell	0.5 μm	Small molecule	Doxorubicin	Capsules loaded with doxorubicin and modified with DR5 are 2–3 times more cytotoxic than the capsules without doxorubicin.	pArg/DS	[32]
Shell	3–5 μm	Extract	*Gratiola officinalis* extract	Encapsulated extract kills 100% of SKBR-3 breast cancer cells and 34% of HeLa cervical cancer cells.	PAH/PSS/DS	[68]
Shell	4 μm	Small molecule	Apigenin, ascorbic acid	The encapsulation efficiency is 20% for both apigenin and ascorbic acid. The release rate is 32–35% within 2 h at physiological pH.	PAH/DS	[69]
Shell	0.25–0.5 μm	Small molecule	Gemcitabine, clodronate	Gemcitabine and clodronate encapsulated in biodegradable polymer multilayer capsules effectively target lung cancer.	pArg/DS	[70]
Shell	3.3–4.8 μm	Protein	BSA, chymotrypsin, lysozyme	α-Chymotrypsin retains ~85% of its original enzymatic activity upon encapsulation.	PAH/PSS	[71]
Shell	5.0–8.3 μm	Small molecule	Tetracycline hydrochloride	Polymer microcapsules have a pectin loading capacity greater than 220 mg/g.	PAH/pectin	[72]
Shell	5.0 μm	Cells	*Escherichia coli*	After encapsulation, ~40% of the cells remain alive.	PAH/PSS	[73]
Shell	4.5 μm	Small molecule	Doxorubicin, nimbin	The IC_50_ for THP-1 cells are 75 and 1.8 µM for nimbin and doxorubicin, respectively. Release of the drugs is remotely activated by NIR laser irradiation.	PAH/PMA/NR	[74]
Shell	5.0 μm	Small molecule	Vitamin B12	Nanoengineered polymer capsules and soft lipid nanovectors are effective carriers for vitamin B_12_.	PAH/PSS	[75]
Shell	5.0 μm	Protein	BSA	A high concentration of NaCl causes considerable dissociation of poly(allylamine) (PAH), apparently due to the action of ionic force.	PAH/PSS	[76]
Shell	4.2–6.3 μm	-	-	A method of surface activation of microcapsules containing the monoclonal antibody trastuzumab has been developed.	PAH/PSS/QD	[36]
Shell	3–4 μm	Protein	Ovalbumin, horseradish peroxidase	Lyophilized microcapsules are candidate adjuvants for antigen delivery; higher immune activation in both in vitro and in vivo assays compared to free antigen has been shown.	pArg/DS	[77]
Shell	3 μm	Nucleic acid	G-quadruplex DNA, double stranded DNA	Poly(methacrylic acid)/poly(N-vinylpyrrolidone) (PMAA/PVPON)_n_ multilayer hydrogel capsules can encapsulate and release ~450 kDa double-stranded DNA.	PMA/PVPON	[29]
Shell	0.65, 3.3 μm	Nucleic acid	mRNA, siRNA	Submicrometer-sized polymer capsules are more efficient in transferring siRNA than micrometer-sized ones used for eGFP mRNA transport.	pArg/DS	[78]
Shell	∼3 μm	Nucleic acid	mRNA, pDNA, plasmid	Microcarriers mediate more efficient transfection than a commercially available liposome-based transfection reagent (>70% vs. <50% for mRNA, >40% vs. 20% for plasmid DNA).	pArg/DS/SiO_2_	[79]
Shell	1–4 μm	Fluorescent dye	Tetramethylrhodamine dextran	Among the four types of DNA capsules studied, the smallest ones with the most integral DNA envelope exhibit the lowest leakage, highest affinity for ATP, and better kinetics and trigger sensitivity.	PAH/DNA	[80]
Shell	2.84 μm	Labeled protein	BSA-Cy7	The localization efficiency of the fluorescent dye in the target kidney after intra-arterial administration is 9 times higher than that in the other kidney and after intravenous injection. After 24 h, no microcapsules are observed in the target kidney.	pArg/DS	[81]
Shell	3–5 μm	Small molecule	Doxorubicin	Microcapsules intensely concentrate positively charged doxorubicin, subsequently releasing it in a controlled manner to effectively induce apoptosis of HepG2 tumor cells.	Chitosan/alginate	[82]
Shell	3–6 μm	Labeled protein	FITC-BSA	Substantially more BSA is loaded at pH 3.8 than at pH 5.0 (i.e., the pI of BSA), and the release of BSA is faster at a higher pH.	PLL/CS	[83]
